# Delirium awareness and care practices among Western European healthcare professionals: a survey

**DOI:** 10.1007/s41999-026-01436-8

**Published:** 2026-02-23

**Authors:** Christoph Leinert, Magnhild Dejgaard, Isabella Glaser, Christian Myrstad, Per R. Nordnes, Marco Salvi, Sylvie Bonin-Guillaume, Barbara C. van Munster, Federico Triolo

**Affiliations:** 1 Institute for Geriatric Research, Ulm University Medical Center at Agaplesion Bethesda Ulm, Zollernring 26, 89073 Ulm, Germany; 2 Department of Geriatric Medicine, Oslo University Hospital, Oslo, Norway; 3 University Department of Geriatric Medicine FELIX PLATTER, Basel, Switzerland; 4 Department of Medicine, Levanger Hospital, Nord-Trøndelag Hospital Trust, Levanger, Norway; 5 Department of Geriatrics and Internal Medicine, Sørlandet Hospital HF, Arendal, Norway; 6 Geriatric Clinic Unit, University Hospital of Parma, Parma, Italy; 7 Department of Medicine and Surgery, University of Parma, Parma, Italy; 8 Service de Médecine Interne-Gériatrie, Assistance Publique Des Hôpitaux de Marseille, Hôpital Nord, Marseille, France; 9 Division of Geriatric Medicine, Department of Internal Medicine, University Medical Center Groningen, University of Groningen, Groningen, the Netherlands; 10 Aging Research Center, Department of Neurobiology, Care Sciences and Society, Karolinska Institutet and Stockholm University, Stockholm, Sweden; 11 Department of Psychiatry, Amsterdam UMC, Vrije University, Amsterdam, The Netherlands

**Keywords:** Delirium, Health care professionals, Survey, Knowledge, Awareness, Geriatrics

## Abstract

**Purpose:**

Despite its high prevalence, delirium remains under-recognized and inadequately managed, with notable differences across clinical and geographical settings. This study aimed to assess current practices, perceived knowledge and barriers to delirium management, and educational preferences among healthcare professionals (HCP) in four Western European countries.

**Methods:**

A cross-sectional online survey was conducted between September 2024 and February 2025 across six hospitals in Norway, Switzerland, Germany, and Italy. Doctors, nurses and therapists (physio-, occupational, and speech-language therapists) from surgical, medical, geriatric, and rehabilitation wards were invited to participate via institutional networks. The survey assessed perceived knowledge on delirium, use of screening tools, implemented prevention strategies, barriers, and preferred educational methods. Data was analyzed using descriptive statistics.

**Results:**

529 HCP responded (72% female; nurses 41.6%, doctors 36.3%). Delirium was frequently encountered, particularly in geriatric and rehabilitation wards; however, 53% reported not using formal assessments, 22% reported using 4AT and 16% CAM. Perceived barriers for better care included lack of time and staffing (69%), limited knowledge (55%), and absence of standard operating procedures (30%). The implementation of preventive strategies and perceived competence varied across settings and countries, with more comprehensive approaches reported in environments with a stronger geriatric focus. Case-based learning emerged as the most preferred educational format across all professional groups (65.5%).

**Conclusion:**

The survey highlights still existing gaps in the implementation of optimal delirium care across different European settings from the HCP perspectives and shows that profession- and context-specific educational initiatives are warranted and might contribute to improvement in care.

**Supplementary Information:**

The online version contains supplementary material available at 10.1007/s41999-026-01436-8.

## Key summary points

**Aim:**

This survey investigates current practices, knowledge levels, and barriers to delirium management, as well as educational preferences, among healthcare professionals (i.e., doctors, nurses, therapists) in four Western European countries.

**Findings:**

Even though delirium was perceived a common condition, half of the healthcare professionals reported not to utilize any delirium assessment tools and to frequently meet different barriers to optimal care including time constraints, knowledge gaps, and lack of standardized procedures. Case-based learning was identified as the most preferred educational format across all professional groups.

**Message:**

Targeted educational initiatives, particularly case-based learning, are necessary to improve delirium care across diverse European healthcare settings.

**Supplementary Information:**

The online version contains supplementary material available at 10.1007/s41999-026-01436-8.

## Background

Delirium is a common yet often underdiagnosed condition among hospitalized patients [1, 2], with prevalence estimates ranging between 11 and 29% [3]. It comes with significant burden, both at the individual and healthcare level [4, 5]. Patients experiencing delirium face considerable distress and higher risk for complications, along with long-term risk of developing dementia, frailty, institutionalization and death [6–8]. At the system level, delirium contributes to longer hospital stays, heightened need for nursing home care, and increased healthcare costs [1].

Optimal delirium management comprises early delirium identification to enable targeted treatment of underlying causes as well as implementation of non-pharmacologic preventative and therapeutic strategies, provision of information to affected patients and involvement of relatives during hospitalization and is best delivered in a multidisciplinary team [9]. Multiple European health care professional societies such as the European Delirium Association (EDA), European Geriatric Medicine Society (EuGMS), European Academy of Nursing Science (EANS), Council of Occupational Therapists for European Countries (COTEC), and the International Association of Physical Therapists working with Older People of the World Confederation for Physical Therapy (IPTOP/WCPT), have acknowledged the importance of delirium as a geriatric syndrome requiring a multidisciplinary approach in prevention, diagnosis and treatment [10]. Many national and international guidelines on delirium management across different European countries [11–14] emphasize the need for a well-structured, primarily non-pharmacological, multidisciplinary preventive approach.

Nevertheless, delirium is often poorly managed in many healthcare settings, including acute care hospitals where it is frequently encountered [9]. The lack of knowledge on delirium and the under-use of screening tools contribute to insufficient recognition and diagnosis as well as inappropriate treatment [2]. Professionals caring for patients at risk of developing delirium need systematic training in the use of screening tools, diagnosing, and management [10]. The implementation of educational programs on delirium varies significantly across European countries. Some regions have advanced programs with extensive use of digital tools and inter-professional education, while others rely on traditional lecture-based methods [15]. Yet, comparative data on healthcare professionals’ perceived knowledge on delirium practices in different European countries remain limited.

We conducted a multidisciplinary survey among healthcare professionals (HCP) in four European countries to assess the perceived competence of HCP in the prevention and care of delirium in hospitalized patients and to identify existing barriers to effective management within and across settings.

## Methods

We conducted an anonymous online survey among different members of the multidisciplinary team (MDT) including doctors, nurses, therapists (i.e., physiotherapist, occupational therapists, speech and language therapists) working in surgical, medical, geriatric and rehabilitation wards of six hospitals in four Western European countries: Norway (Oslo University Hospital, Oslo; Levanger Hospital, Nord-Trøndelag Health Trust; Sørlandet Hospital HF, Arendal), Switzerland (Department of Geriatric Medicine FELIX PLATTER, Basel), Germany (Agaplesion Bethesda Ulm) and Italy (University Hospital Parma). The Norwegian and Italian settings can be characterized as general hospitals with different departments including Geriatrics, while the German and Swiss settings are specialized stand-alone geriatric departments that only run geriatric beds. Table 1 provides a detailed overview of the participating hospitals including hospital type, hospital size, ward size, Intensive care unit size, size of the geriatric department and employed geriatricians.

**Table 1:** Descriptive characteristics by nation

		Total	Norway	Switzerland	Germany	Italy
		N = 529	N = 154	N = 208	N = 66	N = 101
Gender	Male	91 (27.1%)	8 (12.9%)	66 (31.7%)	17 (25.8%)	-
	Female	242 (72.0%)	54 (87.1%)	142 (68.3%)	46 (69.7%)	-
	Diverse	3 (0.9%)	0 (0.0%)	0 (0.0%)	3 (4.5%)	-
Profession	Nurse	220 (41.6%)	71 (46.1%)	78 (37.5%)	19 (28.8%)	52 (51.5%)
	Doctor	192 (36.3%)	63 (40.9%)	65 (31.3%)	21 (31.8%)	43 (42.6%)
	Therapist	117 (22.1%)	20 (13.0%)	65 (31.3%)	26 (39.4%)	6 (5.9%)
Work Experience	< 2 years	56 (12.8%)	9 (14.5%)	42 (20.2%)	5 (7.6%)	9 (8.9%) (< 5 years)
	2–5 years	60 (13.7%)	7 (11.3%)	29 (13.9%)	15 (22.7%)	
	5–10 years	85 (19.5%)	11 (17.7%)	34 (16.3%)	18 (27.3%)	22 (21.8%)
	> 10 years	236 (54.0%)	35 (56.5%)	103 (49.5%)	28 (42.4%)	70 (69.3%)
Workplace	Surgical ward	65 (12.3%)	33 (21.6%)	0 (0.0%)	0 (0.0%)	32 (31.7%)
	Medical ward	117 (22.2%)	69 (45.1%)	0 (0.0%)	0 (0.0%)	48 (47.5%)
	Geriatric ward	218 (41.3%)	38 (24.8%)	120 (57.7%)	45 (68.2%)	15 (14.9%)
	Rehabilitation ward	121 (22.9%)	13 (8.5%)	88 (42.3%)	14 (21.2%)	6 (5.9%)
	Other	7 (1.3%)	0 (0.0%)	0 (0.0%)	7 (10.6%)	0 (0.0%)
Settings	Hospital type		a) & b) University hospital c) University affiliated	University hospital	University affiliated	University hospital
	Hospital size (beds nr)		a) 1778 b) 203 c) 174	300	110	1067
	Ward size (approx. bed nr)		a) 24 b) 8 c) 7	30	30	31
	Geriatric department size (acute care/ rehabilitation bed nr)		a) 21/21 b) 6/0 c) 12/0	130/130	90/20	63/0
	Employed Geriatricians (nr)		a) 17 b) 3 c) 3	12	6	11

Norwegian hospitals include a) University Hospital, Oslo b) Levanger Hospital, Nord-Trøndelag c) Sørlandet Hospital, Arendal. Number of Missing: Gender (n = 193); Work Experience (n = 92); Workplace (n = 1). For technical reasons, the questions for Gender and Work Experience were not asked in one of the Norwegian Hospital (n = 92).

The survey was promoted through intranet and email announcements, dissemination of QR codes on flyers and posters as well as short presentations in shift handovers and personal communication to promote the survey among a broad audience of potential participants with a special focus on geriatric teams including those with limited access to digital information. The survey collection was open for participation for 4 to 6 weeks and was conducted between September 2024 and February 2025. To enhance participation reminder emails were sent on a regular basis.

In every setting approval of the local authorities of the respective institution (chief medical/nursing/therapist officer, data privacy officer) was obtained. The questionnaire had to be adapted in the Italian settings due to privacy concerns regarding the questions about (I) gender and (ii) years of working experience. As no patient data was obtained and the survey among HCP was anonymous, local ethic committees waived the need for ethical approval. Participants were informed about the background and planned analysis of the survey prior to participation and implicitly consented to the further analysis of their responses through their completion of the questionnaire.

The questionnaire consisted of 13 questions and was developed by the authors through an iterative process to balance question length, depth of responses, and feasibility in a healthcare setting. Supplementary file S1 shows the English version of the survey. Questions concerning perceived knowledge were asked as nominal 4 or 5 item Likert scales e.g. risk factor for delirium are: hardly/not known—partly known—known—known and assessed routinely. Question one to five assessed participants background information. Question six and seven evaluated perceived knowledge on delirium. Question eight to eleven identified strategies to prevent and treat delirium established in the respective working environment with a focus on the multidisciplinary non-pharmacologic measures that are known useful in prevention as well as treatment of delirium, e.g. standard operating procedures (SOP) or other local guidelines/protocols for delirium care. The last two questions explored barriers that hinder delirium management and the educational formats healthcare professionals prefer for learning about delirium. Based on the English version initially developed by the authors, one group member translated the survey in their native language (i.e., Norwegian, German, Italian), after which the translation was reviewed by other authors who were native speakers of the same language. The survey was transferred to an online survey format depending on the local regulatory requirements and supported information technology (IT) infrastructures of each setting. Google forms®, Microsoft forms® and Inquery® were used as online survey tools.

### Statistical analysis

Survey responses were translated back into English and harmonized into one dataset. Descriptive statistics and graphical representations were generated for the total sample as well as for national subgroups and ward settings. The datasets were obtained in Microsoft Office (Excel)® and analyzed in STATA 17®.

This initiative was conducted in the context of the fifteenth edition of the European Academy for Medicine of Ageing (EAMA) advanced postgraduate course in geriatrics. EAMA is an institution supported by EuGMS to foster collaborative research and leadership in geriatric medicine (http://www.eama.eu).

## Results

The survey included a total of 529 participants from six hospitals across four Western European countries: Switzerland, Germany, Norway, and Italy. Data from Norwegian hospitals were analyzed together, based on the assumption that hospitals within the same country share similar characteristics and practices reflecting national guidelines and health care systems policies.

### Professional background, clinical settings, and experience

Table 1 summarizes the demographics and professional background of the survey participants and the hospitals by country. Across the full sample, most respondents were female (72.0%), with the proportion highest in Norway (87.1%) and lowest in Switzerland (68.3%). The most common professional groups were nurses (41.6%) and doctors (36.3%), followed by therapists (22.1%). Switzerland and Germany had higher shares of therapists, while Italy had the highest proportion of nurses.

Over half of the participants had more than 10 years of work experience (54.0%), with this share especially high in Italy (69.3%) and Norway (56.5%). Respondents from Switzerland had a more mixed experience profile, with a higher proportion of early-career professionals. In terms of workplace setting, geriatric (41.3%) and rehabilitation wards (22.9%) were most common overall. Norway had the highest proportion in medical wards (45.1%), while Italy had the highest in surgical wards (31.7%) and Switzerland in rehabilitation wards (42.3%). Germany's respondents were mostly concentrated in acute geriatric care (68.2%).Overall hospital size varied between 1778 and 110 beds, with ward sizes between 6 to 31 beds and with 6 to 260 beds run by the geriatric department with 3 to 17 geriatricians employed. All hospitals were formal University hospitals or University affiliated hospitals.

### Perceived contact with patients with delirium

**Fig. 1:**
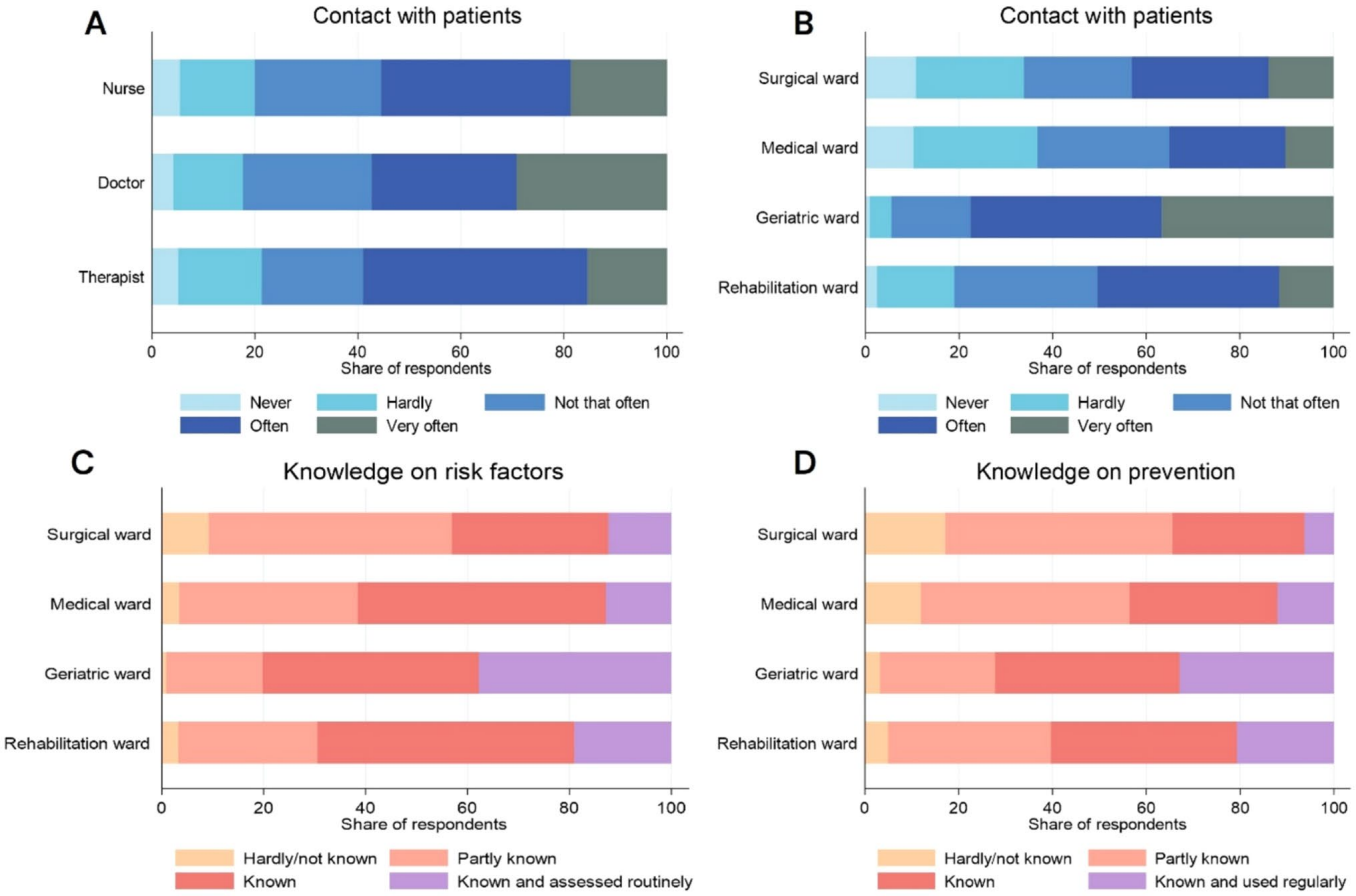
Perceived contact with delirious patients and knowledge on risk factors and prevention. Based on survey question 5 “During my work I have contact with delirious patients ….” by profession (**A**) and clinical setting (**B**). Survey question 6 and 7 “The risk factors of delirium are….” (**C**) and “Strategies to prevent delirium are….” (**D**) by clinical setting

Respondents frequently encountered patients with delirium, with most reporting contact either “often” (n = 186, 35%) or “very often” (n = 115, 22%). Doctors were the most likely to report very frequent contact, followed by nurses, while therapists reported less consistent exposure, with fewer indicating contact “very often” (Fig. 1** A**).

Contact with patients with delirium also varied across clinical settings (Fig. 1** B**). Geriatric and rehabilitation wards had the highest frequency of reported contact, with a large majority indicating that patients with delirium were observed often or very often. In contrast, those working in surgical and medical wards reported lower frequency, with a higher share selecting “hardly” or “not that often.”

### Knowledge on delirium risk factors and prevention/treatment strategies implementation

Most respondents perceived their knowledge on delirium risk factors as *known* (n = 233, 44%) or *known and assessed routinely* in clinical practice (n = 129, 24%), while fewer respondents reported similar familiarity with treatment strategies (*known*, n = 191, 36%; *known and used regularly*, n = 115, 22%). Respondents working in acute geriatric care and geriatric rehabilitation wards reported higher levels of knowledge on delirium risk factors and on prevention/treatment strategies than those in medical or surgical wards (Fig. 1 C and D). Surgical settings reported less knowledge on prevention and risk factors.

Years of professional experience further shaped the patterns of results regarding knowledge on delirium (Supplementary file S2 A and B). Those with more than 10 years of experience were most likely to report both comprehensive understanding and regular use of delirium prevention and treatment strategies. In contrast, respondents with fewer than 5 years of experience—especially those with less than 2 years—were more likely to indicate only limited knowledge.

### Instruments used in clinical practice

Just over half of the respondents (52.9%) reported not using any specific instrument to assess delirium in their clinical practice. Among those who did, the 4AT [16] was the most commonly used tool overall (21.7%), followed by the Confusion Assessment Method (CAM, 16.3%) [17] and the Delirium Observation Screening Scale (DOSS, 8.3%) [18]. The CAM-ICU [19], Nu-DESC [20], and other tools were used by only a small minority.

### Which patients are tested for delirium?

Most of respondents reported to test delirium based on clinical suspicion especially in hyperactive/aggressive patients (70.1%) as well as in hypoactive/sleepy patients (54.1%) or in people with dementia (28.4%). Only a few respondents reported to screen for delirium every day (7.8%) or every shift (4.2%), while screen on admission was more frequently reported (43.1%), especially in the specialized geriatric departments in Germany (57%) and Switzerland (62%). All detailed results are presented in Supplementary file S3.

### What strategies are implemented to prevent and treat delirium

Participants reported using a variety of strategies to manage and prevent delirium in patient care, with variations across countries (Table 2). The most frequently implemented strategies included pain assessment and treatment (69.0%), early mobilization (62.4%), caregiver participation (60.7%), provision of hearing aids or glasses (60.5%), and medication review (59.4%). Sedative medication (52.6%) and reorientation techniques (50.9%) were also commonly used. Concerning general strategies implemented in the respective working environment (Table 3), around half of respondents reported being aware of instructions provided for HCP, e.g. standard operating procedures (43.5%). Only a minority had received a training on delirium in the past 12 months (37.0%). Educational materials on delirium were available for patients and caregivers in approximately 1/4 of settings (26.6%). Moreover, only some patients were informed about delirium risk before elective procedures (21.0%), while documentation of incident delirium in discharge letters was more common (39.8%).

**Table 2:** Implemented strategies to prevent and treat delirium in patients

	Total	Norway	Switzerland	Germany	Italy
	N = 529	N = 154	N = 208	N = 66	N = 101
Providing hearing aids/glasses	320 (60.5%)	119 (77.3%)	146 (70.2%)	37 (56.1%)	18 (17.8%)
Early mobilization	330 (62.4%)	107 (69.5%)	134 (64.4%)	44 (66.7%)	45 (44.6%)
Giving sedative medication	278 (52.6%)	57 (37.0%)	122 (58.7%)	46 (69.7%)	53 (52.5%)
Pain assessment and treatment	365 (69.0%)	108 (70.1%)	163 (78.4%)	41 (62.1%)	53 (52.5%)
Participation of caregivers	321 (60.7%)	96 (62.3%)	137 (65.9%)	36 (54.5%)	52 (51.5%)
Reorientation	269 (50.9%)	104 (67.5%)	113 (54.3%)	34 (51.5%)	18 (17.8%)
Review of medication	314 (59.4%)	99 (64.3%)	132 (63.5%)	44 (66.7%)	39 (38.6%)
Involving of a delirium expert	136 (25.7%)	11 (7.1%)	98 (47.1%)	7 (10.6%)	20 (19.8%)
Other	23 (4.3%)	12 (7.8%)	10 (4.8%)	0 (0.0%)	1 (1.0%)
I don't know	18 (3.4%)	5 (3.2%)	0 (0.0%)	0 (0.0%)	13 (12.9%)

Based on survey question 9: “The following measures are taken regularly to prevent and/or treat delirium in my working environment (more than one answer possible)”.

**Table 3:** Implemented strategies to prevent and treat delirium in the working environments

	Total	Norway	Switzerland	Germany	Italy
	N = 529	N = 154	N = 208	N = 66	N = 101
Instructions for professionals	230 (43.5%)	79 (51.3%)	101 (48.6%)	24 (36.4%)	26 (25.7%)
Information materials for patients and caregivers	141 (26.6%)	18 (11.7%)	93 (44.7%)	23 (34.8%)	7 (6.9%)
In the last 12 month at least 1 training	196 (37.0%)	38 (24.7%)	115 (55.3%)	26 (39.4%)	17 (16.8%)
Delirium in discharge information	210 (39.8%)	12 (7.8%)	137 (65.9%)	29 (43.9%)	32 (31.7%)
Patients are informed on delirium risk before elective procedures	111 (21.0%)	17 (11.1%)	67 (32.2%)	10 (15.2%)	17 (16.8%)
Other	10 (1.9%)	3 (2.0%)	0 (0.0%)	0 (0.0%)	0 (0.0%)

Based on survey question 11: “The following strategies to identify, prevent and treat delirium are established in my working environment”.

Switzerland reported the highest overall use of preventive and treatment strategies, particularly for pain assessment, medication review, early mobilization, and caregiver participation but only half of participants were aware of instructions for HCP and had a training on delirium in last year. Norway emphasized non-pharmacological approaches such as provision of sensory aids, mobilization, and reorientation, but also included pain evaluation and treatment. Most respondents were aware of instructions for HCP but hardly any patient/caregiver information was provided or information on delirium documented in discharge letters. Germany displayed balanced use of both pharmacological and non-pharmacological strategies, while Italy reported the lowest levels of implementation across most categories, with nonetheless relatively higher use of pain management, sedative medication, information on delirium in discharge letters and involvement of caregivers. Involvement of a delirium expert was only frequently reported in Switzerland.

### Reported barriers to delirium management

Table 4 summarizes reported barriers to delirium management across countries. The most frequently cited barrier overall was a lack of time and human resources (69.0%), particularly in Norway, Switzerland, and Germany (over 75% each), while Italy reported this less often (38.6%). Lack of knowledge on delirium was consistently reported at moderate levels across countries (around 55%). Italy more often cited the absence of standard procedures (SOP) (47.5%) and disinterest from doctors and nurses (22.8% and 20.8%, respectively), while these were less perceived as major barriers in the other countries. Switzerland and Norway highlighted environmental limitations, while Germany reported fewer issues overall. Notably, only Switzerland and Italy had a substantial proportion (5.8% and 9.9%) reporting no barriers, while Norway and Germany had none.

**Table 4:** Barriers preventing appropriate provision of care to patients with delirium by nation

	Total	Norway	Switzerland	Germany	Italy
	N = 529	N = 154	N = 208	N = 66	N = 101
Lack of time and human resources	365 (69.0%)	117 (76.0%)	158 (76.0%)	51 (77.3%)	39 (38.6%)
Lack of knowledge on delirium	293 (55.4%)	84 (54.5%)	118 (56.7%)	40 (60.6%)	51 (50.5%)
Lack of interest of doctors	84 (15.9%)	21 (13.6%)	36 (17.3%)	4 (6.1%)	23 (22.8%)
Lack of interest of nurses	85 (16.1%)	19 (12.3%)	28 (13.5%)	17 (25.8%)	21 (20.8%)
Lack of standard operating procedures	158 (29.9%)	47 (30.5%)	47 (22.6%)	16 (24.2%)	48 (47.5%)
Lack of appropriate environment/materials	201 (38.0%)	79 (51.3%)	65 (31.3%)	18 (27.3%)	39 (38.6%)
No barriers, delirium management is optimal	22 (4.2%)	0 (0.0%)	12 (5.8%)	0 (0.0%)	10 (9.9%)
Other	33 (6.2%)	5 (3.2%)	24 (11.5%)	2 (3.0%)	2 (2.0%)

Based on survey question 12 “What barriers can you identify in your working environment that hinder delirium management (more than one answer possible)?”.

### Preferred educational approaches

**Fig. 2:**
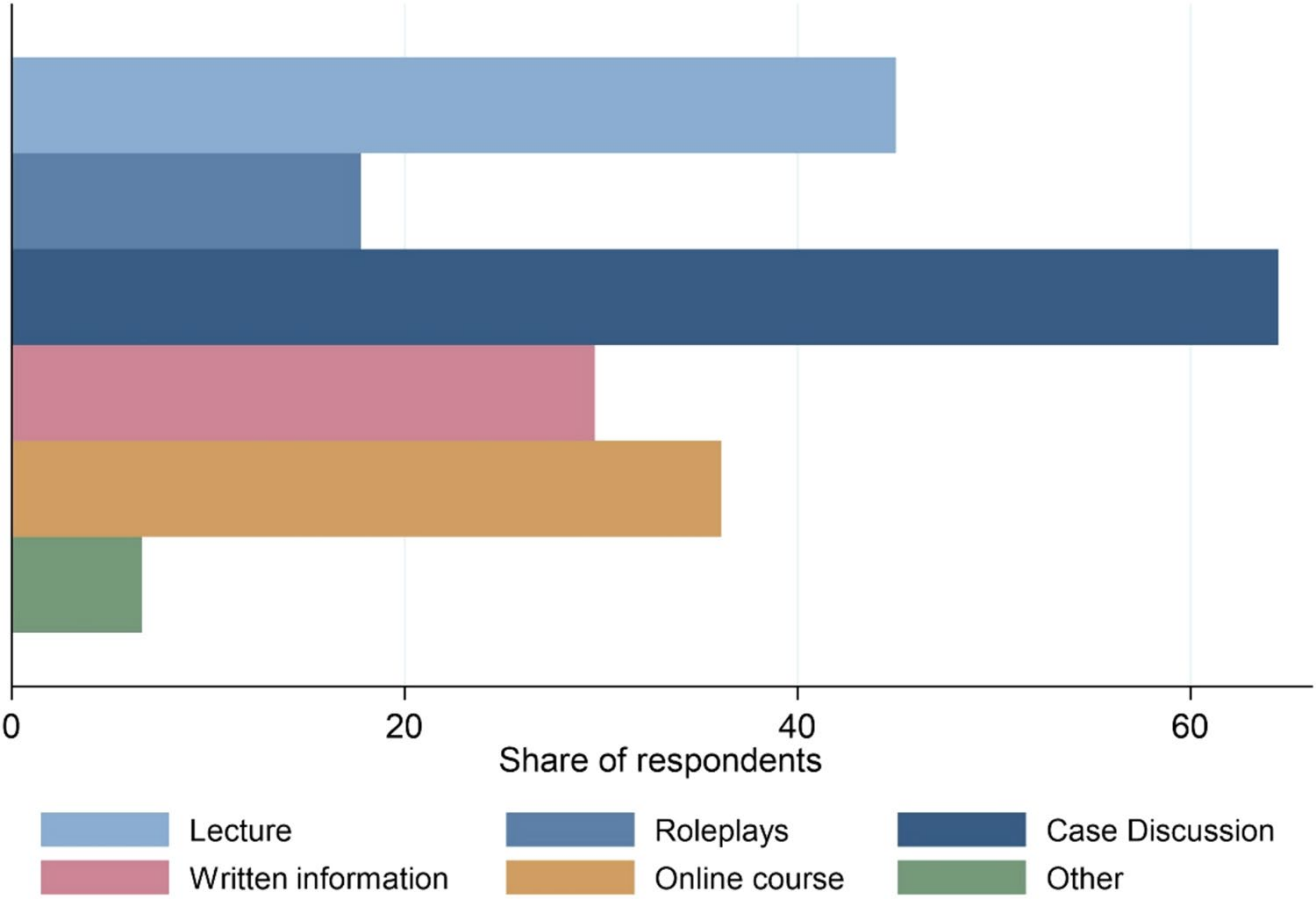
Preferred ways of education on delirium. Based on survey question 13 “If you could choose: Which would be the best way to educate you and your colleagues on the topic of delirium (more than one answer possible)?”

The most preferred educational method was discussion of case examples (64.5%), followed by lectures (45%) and online learning courses (36.1%) (Fig. 2 and Supplementary file S4 for stratification by profession). Case-based learning was particularly favored by doctors (70.3%) and therapists (64.1%), while therapists showed the highest interest in formats like lectures (55.6%) and online courses (49.5%). Written information (29.7%) and roleplay scenarios (17.8%) were less commonly preferred, though slightly more popular among nurses (27.3% and 21.4%). The proportion was similar across all countries (data not shown).

## Discussion

This Western European multidisciplinary survey provides evidence on healthcare professionals’ knowledge and implementation of delirium management strategies in clinical practice. It highlights areas of evidence-based delirium care that are already well established, while also identifying relevant gaps, barriers to implementation, and opportunities for improvement. Taken together, the findings suggest that settings with the most frequent contact with acute geriatric patients, particularly geriatric wards show the greatest awareness and uptake of standard delirium diagnosis and management practices, the least perceived awareness was found in surgical settings. Moreover, the survey sheds light on current clinical practices from the perspective of healthcare professionals and underscores a clear preference for case—discussion based educational formats, which may serve as an effective foundation for targeted quality improvement initiatives.

Most HCPs (57%) reported encountering patients with delirium often or very often and their perceived knowledge of delirium risk factors as well as prevention and treatment strategies as well known or known and regularly applied in clinical practice. Nevertheless, about half (52.9%) of the respondents reported that no validated delirium screening instrument was established in their clinical setting, and that screening was typically conducted when delirium was clinically suspected, particularly in hyper- or hypoactive patients. In comparison, a large multinational survey conducted on World Delirium Awareness Day 2023 reported slightly higher, though still moderate, proportion of respondents (61%) routinely using standardized delirium assessments [21]. Indeed, adherence to routine delirium assessments is known to vary widely, ranging from 19 to 100% across settings [22], which may reflect several factors, including high workload, limited training or the complexity of available tools. Importantly, the use of delirium assessments has been identified as a key marker of the presence of delirium protocols and implementation of management strategies [23], and has been associated with reduced mortality [24]; therefore, it should be actively promoted.

Respondents reported heterogeneous implementation of key elements of delirium prevention and treatment in their working environments. Measures such as pain assessment and treatment (69%) and early mobilization (62.4%) were relatively well established and, even though part of routine care, some delirium specific aspects have to be recognized: For pain management, both uncontrolled pain and the use of centrally acting analgesics (e.g., opioids) are recognized delirium risk factors. Current guidelines emphasize that inadequately treated severe pain represents a greater delirium risk than appropriately administered, opioid-sparing analgesia when indicated [25, 26]. Thus, pain management in geriatric care requires balancing competing risks and often involves targeted clinical decision-making. Regarding mobilization, although early mobilization is widely recommended as part of general care, it is not consistently implemented in routine hospital practice [27]. Moreover, evidence suggests that increased frequency of mobilization beyond usual care, as well as targeted interventions such as evening mobilization, may reduce delirium duration [28, 29]. As we did not distinguish between routine care and delirium specific pain and early mobilization measures, these findings should be interpreted as reflecting the integration of delirium-relevant practices into everyday care rather than delirium-specific interventions. In contrast, other evidence-based strategies, such as structured medication review (59.7%) and reorientation measures (50.9%), were implemented less consistently, despite being key elements of evidence-based non-pharmacological strategies like the Hospital Elder life Program (HELP) or comparable interventions [30–32]. Caregiver involvement, which can facilitate several aspects of non-pharmacological prevention and therapy [33, 34] was reported by 60.7% of respondents. However, only 26.6% provided specific information materials for caregivers. Notably, the use of unfavorable strategies such as sedative medication (52.9%) was reported frequently, a finding consistent with previous surveys [21]. Even though the use of neuroleptics and benzodiazepines in delirium management is not supported by evidence, these medications remain commonly used in many clinical settings, potentially reflecting high staff workload or a lack of implemented non-pharmacological alternatives [35, 36].

Overall, our survey shows a relevant know-do-gap that hinders optimal delirium care, even within settings specialized in geriatric care and those with a strong academic focus, that we consider unsatisfactory. Such discrepancies between knowledge and practice have been well documented in the literature [37], alongside persistent deficits in delirium-related knowledge among healthcare professionals [38, 39]. Insufficient education and training have also been consistently recognized as major barriers to effective delirium management [40]. However, when respondents were asked about barriers to implementing prevention and treatment strategies, the most frequently cited reason was lack of human and/or time resources (69%), a challenge consistently identified in previous studies [41, 42]. This was followed by a lack of knowledge about delirium (55.4%), whereas disinterest among doctors (15.9%) and nurses (16.1%) was reported far less often. The predominance of structural barriers over attitudinal ones suggests that interventions may prioritize resource allocation and workflow redesign, rather than focusing solely on HCP motivation or competence.

To our knowledge, this is the first study to describe which types of educational interventions HCPs value most in relation to delirium. Consistently across all professional groups, discussion of case examples was rated as the most valuable format (64.5%), followed by in-person lectures (45%) and online learning courses (36.1%). Case-based discussion is a common and effective component of education programs. In a review on delirium education programs in inpatient hospital care, such discussions were included in 13 out of 42 programs and were deemed particularly suitable for interprofessional education and professional development [43]. The evidence on e-learning interventions to improve delirium knowledge and care remain mixed. While a study could show relevant improvements in delirium knowledge after a short e-learning intervention [44], other studies have not found significant changes on delirium prevalence, duration or nurses’ knowledge levels [45]. Roleplays or simulation-based learning was least preferred among participants (17.8%), even though simulation has been described as a potentially effective option to teach delirium, at least in undergraduate education [46, 47]. Simulation-based training has also shown to enhance communication skills among HCP, as well as interaction with patients and caregivers in delirium-related contexts [48]. Together, these findings indicate that blended, case-based and interactive formats may be best suited to engage healthcare professionals across disciplines.

Beyond individual education, institutional support also emerged as a critical factor. Only 47.5% of respondents were aware of existing delirium care plans, despite such plans being available in all participating institutions, and 29.9% identified the lack of standard operating procedures a potential barrier. This limited awareness of existing protocols may reflect shortcomings in internal communication or in the accessibility and structure of the protocols themselves [49]. The appointment of a delirium expert, such as an advanced practice nurse specialist, has been shown as an effective way to enhance awareness and adherence to standard operating procedures [50]. However, only a minority of respondents (25.7%) reported that such role had been implemented in their institution. Establishing this kind of dedicated role may also facilitate ongoing support and continuing education to frontline staff, a key element for successful quality improvement projects [43, 51, 52]. The most effective programs tend to combine multiple educational methods to address complementary learning goals of *knowing* (e.g. lectures, online courses), *meaning* (e.g. discussion of case examples) and *doing* (e.g. patient simulation) [46, 53]. Ideally, a setting-specific, continuous educational approach should be established, beginning already at the undergraduate level and continuing throughout postgraduate training for all members of the MDT [43, 46]. Such sustained, institutionally supported strategies may help address the know–do gap identified in this survey to foster healthcare environments in which delirium is optimally managed.

This study has several strengths. It represents a coordinated, multinational effort to gather evidence on healthcare professionals’ perceived knowledge of delirium and educational preferences across hospitals in different European countries. The inclusion of respondents from diverse professional backgrounds, including physicians, nurses, and therapists, adds further value, as it reflects the multidisciplinary nature of delirium care and allows for comparisons across clinical roles. The use of a standardized questionnaire developed through a collaborative network of geriatricians ensured consistency across sites, while translation into national languages enhanced usability and accessibility for respondents in diverse healthcare contexts.

However, some limitations should be acknowledged. The survey covered only four countries, all from Western Europe, and differences in hospital types and departmental structures may limit the comparability of findings across settings. Moreover, as the survey was disseminated online through professional networks, selection bias cannot be excluded, as respondents who are more engaged with professional organizations may not be representative of all healthcare professionals. We also acknowledge as a limitation that we were not able to ask for the specific proportion of geriatricians or other professionals with geriatric specialization as a potentially valuable piece of information from the survey, due to data protection regulations. Further, we asked in combination for prevention and treatment strategies but not separately. As most non-pharmacologic strategies that we focus on in this multidisciplinary survey can be taken as well as prevention and treatment measures [54], we still believe that this is not limiting the validity of the survey’s results. Focusing on the evaluation of potential barriers rather than facilitators might have limited our ability to capture potential benefits of facilitator-based actions on the latter, although barriers and facilitators have often been described as two sides of the same coin [49]. Finally, the study assessed perceived rather than objectively tested knowledge, as the aim was to explore self-reported understanding and practices related to delirium.

## Conclusion

This survey indicates that healthcare professionals’ perceived knowledge of delirium remains suboptimal and that delirium-related practices across European hospitals are still under implemented. These findings highlight the need for focused, context-specific educational initiatives, coupled with the development of dedicated delirium expertise within healthcare teams. Such efforts have the potential to improve the quality of care for hospitalized patients at risk of, or experiencing, delirium. Continued education and reinforcement will be crucial to ensure the sustained effectiveness of these initiatives and to inform future quality improvement strategies that could be monitored by regular surveys of perceived knowledge, barriers and facilitators of optimal delirium care.

## Supplementary Information

Below is the link to the electronic supplementary material.

 Supplementary file S1 (DOCX 19 KB) 

Supplementary file S2 (JPG 182 KB)

Supplementary file S3 (DOCX 16 KB) 

Supplementary file S4 (JPG 69 KB)

## Data Availability

Data from the survey are available on request.
