# Supplementary material for: Delirium awareness and care practices among Western European healthcare professionals: a survey
Source: Eur Geriatr Med. 2026 Feb 23;17(3):1333–44. doi: 10.1007/s41999-026-01436-8 (PMC13309479; doi:10.1007/s41999-026-01436-8)
Supplement: Supplementary file 1 — Supplementary file S1 (DOCX 19 KB) [file 41999_2026_1436_MOESM1_ESM.docx]

**Supplementary file S1: Questions included in the survey.**

| No. | Question | Answer Options |
| --- | --- | --- |
| 1 | What is your gender? | A) Male  B) Female  C) Diverse |
| 2. | What is your profession? | 1. Nurse 2. Doctor 3. Therapist |
| 3. | How long have you been working in this profession? | A) Less than 2 years  B) 2 to 5 years  C) 5 to 10 years  D) More than 10 years |
| 4. | What is your primary working environment? | A) a surgical ward  B) a medical ward  C) a geriatric ward  D) a rehabilitation ward |
| 5. | During my work I have contact with delirious patients …. | A) … very often  B) … often  C) … not that often  D) … hardly  E) … never |
| How would you rate your personal knowledge on delirium? | | |
| 6. | The risk factors of delirium …. | A) … are known and assessed routinely  B) … are known  C) … are partly known  D) … are hardly/not known |
| 7. | Strategies to prevent delirium …. | A) … are known and used regularly  B) … are known  C) … are partly known  D) … are hardly/not known |
| How would you rate the strategies to identify, prevent and treat delirium in your working environment? | | |
| 8. | Are screening instruments used to identify delirium? | A) ....no  B) ….yes, the following: CAM  C) … yes, the following: CAM-ICU  D) … yes, the following: 4AT  E) … yes, the following: Nu-DESC  F) … yes, the following: DOSS  G) … yes, other: ___________ |
| 9. | The following strategies to identify, prevent and treat delirium are established in my working environment. | 1. Instructions for professionals (e.g. SOP)?   ☐ Yes/ ☐ No/ ☐ Don’t know   1. Information materials for patients and caregivers?   ☐ Yes/ ☐ No/ ☐ Don’t know   1. In the last 12 month at least 1 training was held concerning delirium?   ☐ Yes/ ☐ No/ ☐ Don’t know  D) „Delirium“ is part of the discharge information (e.g. discharge letter)  ☐ Yes/ ☐ No/ ☐ Don’t know   1. Patients are informed on delirium risk before elective procedures   ☐ Yes/ ☐ No/ ☐ Don’t know   1. Other:_____________________________ |
| 10. | At what times are patients examined for delirium in your working environment (more than one answer possible)? | A) Never  B) In hyperactive/aggressive patients  C) In hypoactive/sleepy patients  D) On admission on a regular basis  E) In persons with known dementia  F) Once a day  G) Once each shift  H) In other situations/ If other risk factors are recognized: ______________________  I) I don’t know. |
| 11. | The following measures are taken regularly to prevent and/or treat delirium in my working environment (more than one answer possible): | 1. Providing hearing aids/ glasses 2. Early mobilisation 3. Giving sedative medication 4. Pain assessment and treatment 5. Participation of caregivers 6. Reorientation 7. Review of medication 8. Involving of a delirium expert 9. Other: ________________ 10. I don’t know. |
| 12. | What barriers can you identify in your working environment that hinder delirium management (more than one answer possible)? | A) Lack of time and human resources  B) Lack of knowledge on delirium  C) Lack of interest of doctors  D) Lack of interest of nurses  E) Lack of standard operating procedures (SOPs)  F) Lack of appropriate environment/materials  G) No barriers, delirium management is optimal  H) Other: _____________________________ |
| 13. | If you could choose: Which would be the best way to educate you and your colleagues on the topic of delirium (more than one answer possible)? | A) Lecture  B) Roleplays on the topic  C) Discussion of case examples  D) Written information  E) Online learning course  Other: _________________________________ |
