# Supplementary figures and images for: Delirium awareness and care practices among Western European healthcare professionals: a survey
Source: Eur Geriatr Med. 2026 Feb 23;17(3):1333–44. doi: 10.1007/s41999-026-01436-8 (PMC13309479; doi:10.1007/s41999-026-01436-8)

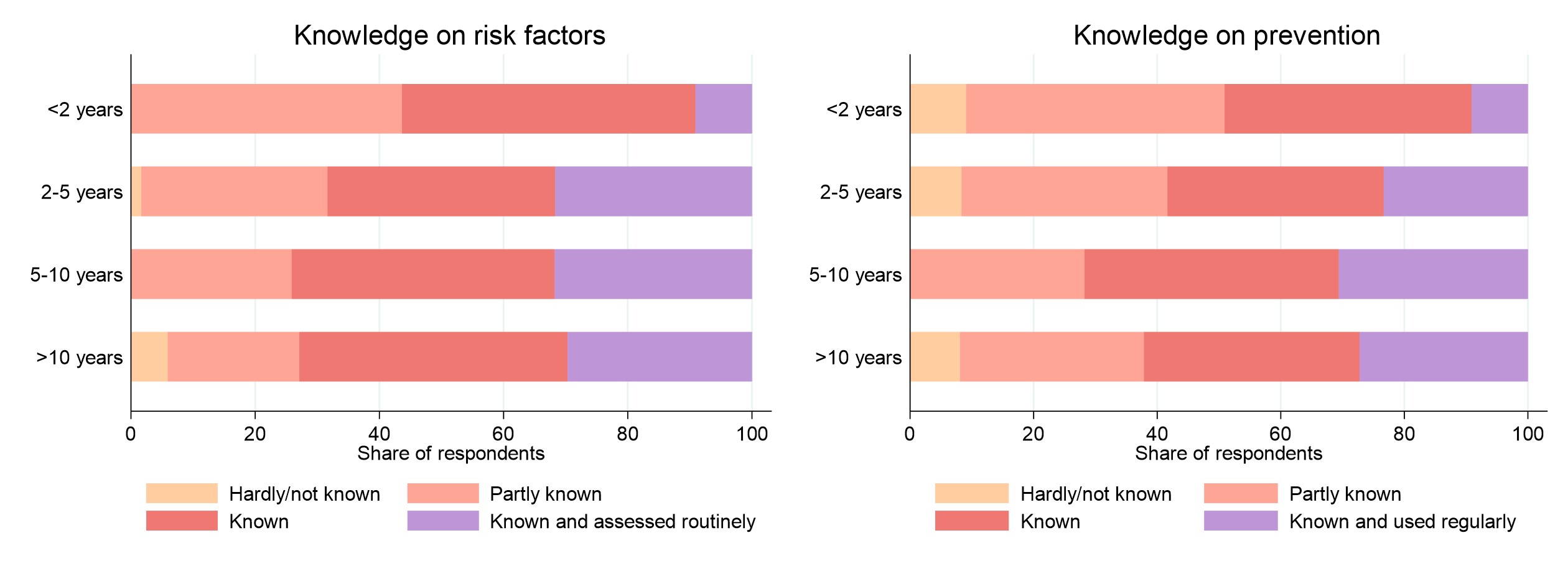

Supplement: Supplementary file 2 — Supplementary file S2 (JPG 182 KB) [file 41999_2026_1436_MOESM2_ESM.jpg]

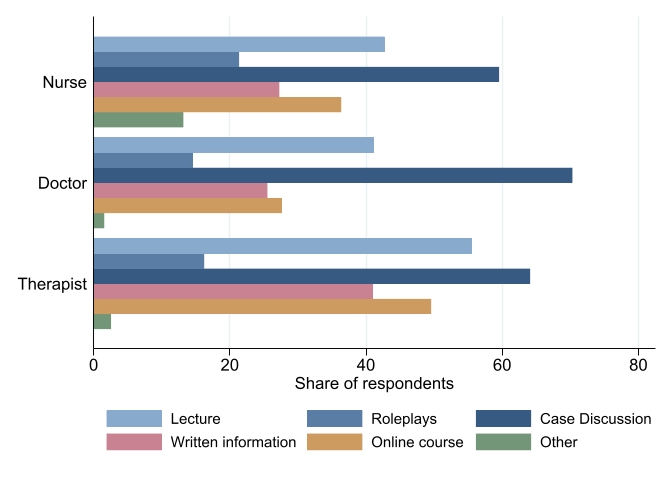

Supplement: Supplementary file 4 — Supplementary file S4 (JPG 69 KB) [file 41999_2026_1436_MOESM4_ESM.jpg]
