# Supplementary material for: Delirium awareness and care practices among Western European healthcare professionals: a survey
Source: Eur Geriatr Med. 2026 Feb 23;17(3):1333–44. doi: 10.1007/s41999-026-01436-8 (PMC13309479; doi:10.1007/s41999-026-01436-8)
Supplement: Supplementary file 3 — Supplementary file S3 (DOCX 16 KB) [file 41999_2026_1436_MOESM3_ESM.docx]

**Supplementary file S3: Delirium screening practices stratified by country**. Based on the survey question 10: “At what times are patients examined for delirium in your working environment (more than one answer possible)?”

|  | **Total** | **Norway** | **Switzerland** | **Germany** | **Italy** |
| --- | --- | --- | --- | --- | --- |
|  | N=529 | N=154 | N=208 | N=66 | N=101 |
| **Never** | 39 (7.4%) | 2 (1.3%) | 14 (6.7%) | 6 (9.1%) | 17 (16.8%) |
| **In hyperactive/aggressive patients** | 371 (70.1%) | 125 (81.2%) | 151 (72.6%) | 35 (53.0%) | 60 (59.4%) |
| **In hypoactive/sleepy patients** | 286 (54.1%) | 119 (77.3%) | 119 (57.2%) | 27 (40.9%) | 21 (20.8%) |
| **On admission on a regular basis** | 228 (43.1%) | 43 (27.9%) | 129 (62.0%) | 38 (57.6%) | 18 (17.8%) |
| **In persons with known dementia** | 150 (28.4%) | 28 (18.2%) | 75 (36.1%) | 15 (22.7%) | 32 (31.7%) |
| **Once a day** | 41 (7.8%) | 19 (12.3%) | 16 (7.7%) | 1 (1.5%) | 5 (5.0%) |
| **Once each shift** | 22 (4.2%) | 3 (1.9%) | 8 (3.8%) | 1 (1.5%) | 10 (9.9%) |
| **Other** | 100 (18.9%) | 47 (30.5%) | 38 (18.3%) | 5 (7.6%) | 10 (9.9%) |
| **I don't know** | 10 (1.9%) | 5 (3.2%) | 0 (0.0%) | 0 (0.0%) | 5 (5.0%) |
